# Progressive subretinal fibrosis and uveitis syndrome: clinical course and multimodal imaging analysis in a late-onset case

**DOI:** 10.1186/s12348-026-00626-6

**Published:** 2026-08-03

**Authors:** Alessandro Feo, Phillip P. Le, Kirk Hou, Colin A. McCannel, Edmund Tsui, Judy L. Chen

**Affiliations:** 1 Stein Eye Institute, David Geffen School of Medicine at UCLA, Los Angeles, CA USA; 2 Department of Biomedical Sciences, Humanitas University, Via Rita Levi Montalcini 4, 20072 Pieve Emanuele-Milan, Italy; 3 Doheny Eye Institute, Los Angeles, CA USA

**Keywords:** Imaging, Optical coherence tomography, OCT, Subretinal fibrosis and uveitis, SFU, Uveitis

## Abstract

**Purpose:**

To describe the longitudinal clinical and multimodal imaging findings of a patient with progressive subretinal fibrosis and uveitis (SFU) syndrome.

**Case presentation:**

Case report. An 85-year-old woman with a history of cardiomyopathy, systemic hypertension, hyperlipidemia and hypothyroidism presented with a 5-day history of acute bilateral visual decline. At baseline, best-corrected Snellen visual acuity was 20/80 OD and 20/400 OS. Fundus examination revealed multifocal chorioretinal lesions with fibrotic subretinal changes and vitreous inflammation. Fundus autofluorescence demonstrated scattered areas of hypoautofluorescence with hyperautofluorescent borders, and fluorescein angiography revealed window defects with late staining but no leakage. Spectral-domain optical coherence tomography (SD-OCT) showed diffuse retinal pigment epithelium (RPE)-Bruch’s membrane (BrM) splitting and overlying fuzzy subretinal hyperreflective material (SHRM). Primary vitreoretinal lymphoma and multifocal choroiditis were initially suspected; however, systemic and uveitis work-up was negative. The patient’s advanced age was atypical for new-onset SFU and further contributed to diagnostic uncertainty. Despite oral corticosteroid treatment and intravitreal dexamethasone implants OU, the disease progressed relentlessly, leading to widespread subretinal fibrosis and chorioretinal atrophy, and severe visual decline (20/100 OD, counting fingers OS at 12 months).

**Conclusion:**

SFU is a rare, aggressive, and underrecognized immune-mediated uveitic syndrome that can masquerade as neoplastic or inflammatory chorioretinopathies. This atypical late-onset case expands the known age spectrum of the disease and highlights the importance of considering SFU in rapidly progressive chorioretinal fibrosis in older adults. OCT biomarkers – particularly RPE–BrM splitting and fuzzy SHRM – may signal early SFU, emphasizing timely recognition and immunomodulatory therapy to prevent vision loss.

## Introduction

Progressive subretinal fibrosis and uveitis (SFU) or SFU syndrome is an exceptionally rare, aggressive, and potentially underdiagnosed entity, first described by Palestine et al. in 1984 [1].

SFU is characterized by chronic intraocular inflammation with whitish subretinal “fibrotic-like” lesions that progressively enlarge, coalesce, and result in profound vision loss [1, 2]. Histopathological studies have demonstrated retinal gliosis, thick fibrotic tissue derived from the retinal pigment epithelium (RPE), and granulomatous lymphocytic infiltration within the choroid, supporting the hypothesis of an immune-mediated disease process targeting the retina, RPE, and inner choroid [3, 4]. Despite corticosteroid and immunosuppressive therapies, the prognosis remains poor, though recent reports have suggested that intravitreal corticosteroid implants and biologics such as anti-TNF agents may offer disease stabilization in selected cases [5–7].

Although most reported cases have occurred in younger to middle-aged adults, new-onset SFU can also develop in older individuals, underscoring that age alone should not exclude this diagnosis [1–4, 6].

In our report, we describe the clinical course and multimodal imaging follow-up of an 85-year-old female with SFU, underscoring the importance of tomographic findings for early recognition of the disease.

## Case presentation

We conducted a retrospective review of a patient presenting to the UCLA Uveitis Service with bilateral subretinal fibrosis and uveitis. Demographic and clinical data including age, gender, medical and ocular history were collected. Snellen visual acuity (VA), slit-lamp biomicroscopy, and dilated fundus examination were performed at each visit. Multimodal imaging included spectral-domain optical coherence tomography (SD-OCT) (Heidelberg Spectralis OCT; Heidelberg Engineering, Heidelberg, Germany and Cirrus HD-OCT; ZEISS, Germany), ultrawidefield (UWF) color fundus photography, UWF fundus autofluorescence (FAF), and UWF fluorescein angiography (FA) and indocyanine green angiography (ICGA) (Optos California, Marlborough, MA, USA).

An 85-year-old female with a history of cardiomyopathy, systemic hypertension, hyperlipidemia and hypothyroidism was referred to the UCLA Uveitis Service for evaluation of acute on chronic bilateral visual decline. She also had a pre-existing diagnosis of exudative age-related macular degeneration (AMD), for which she had been receiving intravitreal bevacizumab injections in both eyes (OU) at an outside institution prior to referral.

**Fig. 1:**
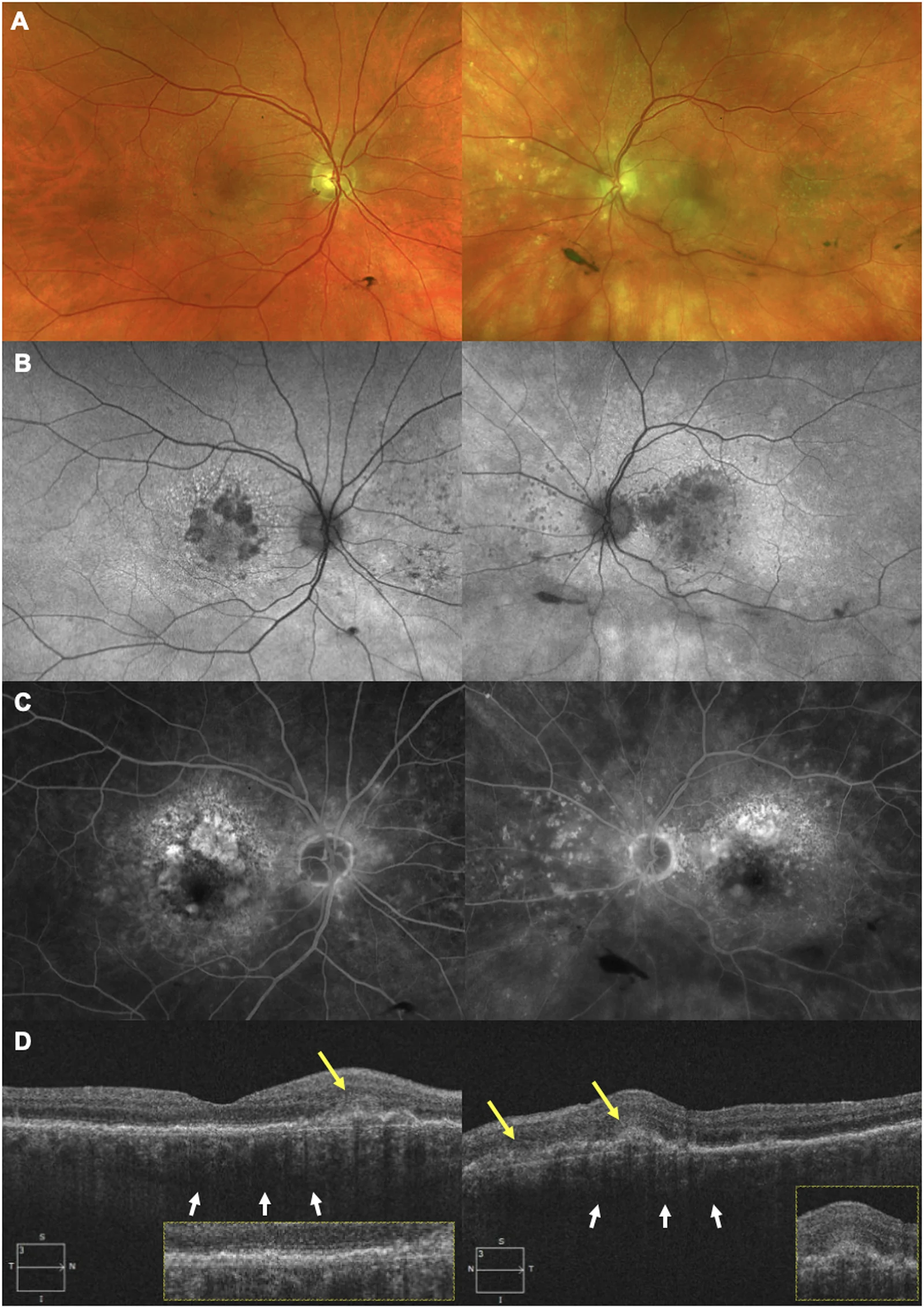
Multimodal imaging of both eyes (OU) of an 85-year-old female patient with progressive subretinal fibrosis and uveitis syndrome at baseline. **(A)** Ultra-widefield (UWF) color fundus photography shows multifocal chorioretinal lesions surrounded by smaller “drusenoid” lesions with subretinal fibrotic changes in the macula OU. **(B)** UWF Fundus autofluorescence illustrates scattered areas of hypoautofluorescence with hyperautofluorescent borders OU. **(C)** UWF Fluorescein angiography demonstrates window defects and late staining of multifocal lesions without vascular leakage OU. **(D)** Optical coherence tomography B-scans show thick choroid (white arrows) and diffuse retinal pigment epithelium-Bruch’s membrane splitting in the macula with fuzzy subretinal hyperreflective material OU (yellow arrows and yellow zoomed rectangles)

At presentation, Snellen VA was 20/80 in the right eye (OD) and 20/400 in the left eye (OS). The anterior segment examination was unremarkable except for the presence of bilateral posterior chamber intraocular lens implants OU. Intraocular pressure was within normal limits OU. Dilated fundus examination (Fig. 1A) revealed multifocal chorioretinal lesions surrounded by smaller “drusenoid” lesions with subretinal fibrotic changes and vitreous inflammation (SUN grade 1 + vitreous cells) OU. FAF (Fig. 1B) demonstrated scattered areas of hypoautofluorescence with hyperautofluorescent borders, while FA (Fig. 1C) showed window defects and late staining of multifocal lesions without vascular leakage OU.

SD-OCT (Fig. 1D) revealed diffuse RPE-Bruch’s membrane (BrM) splitting in the macula with overlying fuzzy subretinal hyperreflective material (SHRM) and a thickened choroid OU.

The patient was initially suspected to have primary vitreoretinal lymphoma (PVRL) due to the presence of sub-RPE infiltrates in the setting of her age and constitutional symptoms of fatigue and weight loss over the preceding months. However, PET and CT of the head and orbits were negative, and oncologic evaluation revealed no evidence of systemic lymphoma. Given the relatively mild vitreous inflammation, bilateral symmetric presentation, and lack of systemic or neuro-ophthalmic evidence suggestive of lymphoma, diagnostic vitrectomy/intraocular biopsy and aqueous or vitreous cytokine analysis (including IL-10/IL-6 ratio assessment) were not pursued. Systemic work-up was negative or within normal limits (rheumatoid factor, anti-neutrophil cytoplasmic antibody, angiotensin converting enzyme, Lyme antibody, treponema pallidum particle agglutination assay, and QuantiFERON Gold), except for an RPR titer of 1:1, of uncertain clinical significance. Therefore, the patient was started on oral prednisone 60 mg daily, which was maintained and gradually tapered over a two-month period.

Multifocal choroiditis (MFC) and progression of underlying AMD were also considered in the differential diagnosis, given the presence of drusenoid-appearing lesions on fundus examination, hypoautofluorescent areas on FAF, and RPE-BrM splitting/separation on OCT, potentially corresponding to basal laminar deposits.

**Fig. 2:**
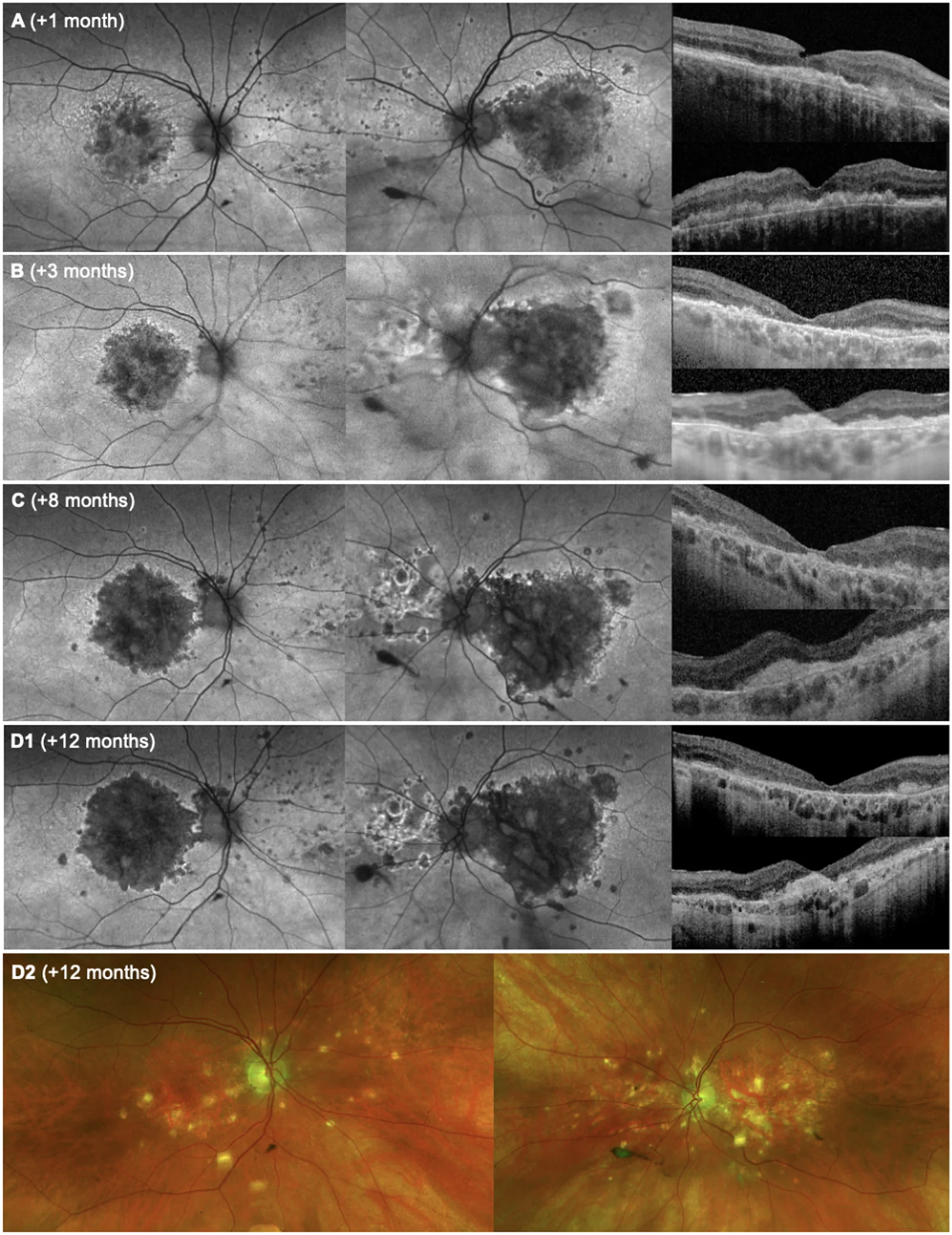
Serial fundus autofluorescence (FAF) and cross-sectional optical coherence tomography (OCT) scans over 12 months of follow-up (A = 1 month; B = 3 month; C = 8 months, D = 12 months, i.e. last available follow-up). OCT scans from the right eye are shown on the top row and OCT scans from the left eye are shown on the bottom row throughout the figure. **A-D1.** FAF and OCT B-scans illustrate the rapid and inexorable progression of bilateral subretinal fibrosis and macular atrophy OU typically associated with progressive subretinal fibrosis and uveitis syndrome. **D2.** Color fundus photography at the last follow-up demonstrates extensive posterior pole atrophy and fibrosis OU

She was subsequently treated with intravitreal dexamethasone 0.7 mg implants in both eyes, which led to partial improvement of vitreous cellular inflammation and haze. The first injection was administered OS three months after presentation, followed by a second injection OS at five months. Additional ICGA imaging demonstrated corresponding hypofluorescent choroidal lesions OU. Systemic immunomodulatory therapy was recommended; however, the patient declined due to advanced age and concerns regarding infection risk. However, despite therapy, the patient developed severe and progressive subretinal fibrosis with widespread chorioretinal atrophy, consistent with progressive SFU syndrome. Serial SD-OCT demonstrated increasing RPE-BrM splitting and SHRM, culminating in confluent subretinal fibrosis and macular atrophy, findings that were corroborated by FAF (Fig. 2) within 12 months of follow-up.

At the last available visit (12 months), VA declined to 20/100 OD and count fingers OS.

## Discussion

Progressive SFU, also referred to as diffuse subretinal fibrosis syndrome, is a rare and aggressive inflammatory disorder of the posterior segment characterized by chronic intraocular inflammation and rapidly progressive whitish subretinal fibrotic plaques [1–3]. Since its first description by Palestine et al. in 1984 [1], SFU has been recognized as a distinct and often devastating uveitic entity, but its nosological classification remains debated.

The hallmark clinical features are vitreous inflammation, multifocal chorioretinal lesions, and the inexorable and rapid progression of bilateral subretinal fibrosis and macular atrophy, often culminating in profound visual loss within months to years [1–3]. Histopathological analyses across multiple reports have consistently demonstrated gliotic retina, thick fibrotic subretinal tissue originating from the RPE, and granulomatous inflammatory infiltration of the choroid with predominance of lymphocytes and plasma cells. Immunopathologic studies further revealed deposition of immunoglobulin and complement along the BrM and infiltration by T cells, B cells, and macrophages arranged in pseudogerminal centers [1, 4].

The 2002 clinicopathologic study by Wang et al. [3] demonstrated circulating autoantibodies directed against the RPE and photoreceptors, and immunohistochemistry showed collagen types III, IV, V, and VI within fibrotic plaques. These findings suggested that SFU may represent an autoimmune process overlapping with, or even constituting a variant of, sympathetic ophthalmia, thereby linking the condition to autoimmune reactivity against previously sequestered ocular antigens. This concept is reinforced by the immunopathology study of Lim et al. [4], which showed heavy chorioretinal infiltration by CD3 + T cells, CD20 + B cells, and CD68 + macrophages.

Clinically, SFU may mimic several posterior uveitis and masquerade syndromes, including MFC, PVRL, and placoid disorders. In our case, the presence of diffuse sub-RPE infiltration and RPE-BrM splitting with fuzzy SHRM [8] raised initial suspicion for PVRL and MFC. However, the bilateral rapid progression to confluent subretinal fibrosis and atrophy, along with the absence of systemic malignancy, favored the diagnosis of SFU. This diagnostic uncertainty was further compounded by the patient’s advanced age, as new-onset SFU typically occurs in younger or middle-aged individuals [1–7], making PVRL a more likely initial consideration in an 85-year-old patient. Importantly, anti-VEGF therapy was discontinued after the onset of inflammatory symptoms and no clear temporal association was identified between intravitreal bevacizumab injections and worsening intraocular inflammation or fibrotic progression. Moreover, the bilateral progressive evolution toward diffuse subretinal fibrosis and chorioretinal atrophy was considered atypical for anti-VEGF–associated sterile inflammation, infectious endophthalmitis/chorioretinitis, or inflammatory sequelae related to intravitreal injections. The absence of retinal vascular leakage, hypopyon, significant anterior chamber inflammation, or focal infectious retinitis further argued against an infectious or injection-related inflammatory process.

Additionally, although the degree of subretinal fibrosis in our case was relatively milder and involved a more limited retinal area (predominantly the posterior pole) than in some of the classically reported SFU cases [1–7], the progressive and bilateral nature of the fibrotic evolution – occurring despite systemic and intravitreal corticosteroid therapy – supports its inclusion within the spectrum of progressive SFU. The case may therefore represent an atypical or early-stage presentation of SFU, where fibrosis remains subclinical or less confluent but demonstrates characteristic tomographic and clinical progression over time. This milder phenotype underscores the variability of disease expression and highlights the importance of recognizing SFU even when subretinal fibrosis appears more limited.

Importantly, recent advances in OCT imaging have identified potential biomarkers that may provide additional diagnostic value in the context of SFU. Diffuse RPE-BrM separation in the macula [9], previously described in severe progressive atrophic maculopathies such as late AMD [10] and, more recently, extensive macular atrophy with pseudodrusen-like appearance (EMAP) – likely reflecting basal laminar deposits – may represent an analogous feature in SFU. However, when observed bilaterally and in association with accelerated subretinal fibrotic evolution, this finding could serve as an early indicator of SFU, reflecting underlying lymphocytic infiltration of the inner choroid [3, 4].

Encouragingly, more recent evidence [5–7] supports a role for biologic therapy targeting TNF-α and B cells. Adán et al. [5] reported a dramatic response to intravenous infliximab injections in a patient refractory to oral prednisone, cyclosporine and azathioprine, while Cornish et al. [6] described stabilization with rituximab, emphasizing the pathogenic contribution of B cells. More recently, Moll-Udina et al. [7] demonstrated long-term control with a combination of intravitreal dexamethasone implants and adalimumab. These reports highlight a paradigm shift in management toward biologics, aligning with the histopathological evidence of both T- and B-cell driven autoimmunity.

Despite these advances, delayed recognition remains the primary obstacle in the management of this rare entity. By the time fibrosis becomes clinically apparent, visual prognosis is often dismal. Our case confirms the poor natural history of SFU, with marked bilateral decline to legal blindness within months despite intravitreal and systemic corticosteroid therapy. Thus, the identification of early multimodal imaging biomarkers – notably RPE-BrM splitting and fuzzy SHRM – may be critical for prompt diagnosis and initiation of more aggressive immunomodulatory therapy.

In conclusion, our report contributes by illustrating characteristic OCT biomarkers of early subretinal fibrosis, that may facilitate prompt identification and treatment of this otherwise rare and blinding disorder. Despite poor response of SFU to corticosteroids and conventional immunosuppressants, biologics such as infliximab, adalimumab, and rituximab appear promising for disease stabilization, although validation in larger cohorts is needed.

## Data Availability

No datasets were generated or analysed during the current study.
